# Conservation Laws and the Philosophy of Mind: Opening the Black Box, Finding a Mirror

**DOI:** 10.1007/s11406-019-00102-7

**Published:** 2019-07-31

**Authors:** J. Brian Pitts

**Affiliations:** Faculty of Philosophy and Trinity College, University of Cambridge, Cambridge, UK

**Keywords:** Conservation laws, Noether’s theorems, Philosophy of mind, Dualism, Cartesianism, Interactionism, Gravitational energy

## Abstract

Since Leibniz’s time, Cartesian mental causation has been criticized for violating the conservation of energy and momentum. (Non-epiphenomenalist property dualism is analogous.) Many dualist responses clearly fail. But conservation laws have important neglected features generally undermining the objection. Conservation is *local*, holding first not for the universe, but for everywhere separately. The energy (or momentum, *etc.*) in any volume changes only due to what flows through the boundaries (no teleportation). Constant total energy holds if the global summing-up of local conservation laws converges; it probably doesn’t in reality. Energy (momentum) conservation holds if there is symmetry, the sameness of the laws over time (space). Thus, if there are time-places where symmetries fail due to nonphysical influence, conservation laws fail there and then, while holding elsewhere, such as refrigerators and stars. Noether’s converse first theorem shows that conservation laws imply symmetries. Thus conservation trivially nearly entails the causal closure of the physical. But expecting conservation to hold in the brain (*without looking*) simply assumes the falsehood of Cartesianism. Hence Leibniz’s objection begs the question. Empirical neuroscience is another matter. So is Einstein’s General Relativity: far from providing a loophole, General Relativity makes mental causation *harder*.

## Introduction

The energy conservation objection to nonphysical mental causation has been made from the 1690s (Leibniz 1997) to the 2010s. According to Leibniz’s *Theodicy*,

…two important truths on this subject have been discovered since M. Descartes’ day. The first is that the quantity of absolute force which is in fact conserved is different from the quantity of movement, as I have demonstrated elsewhere. The second discovery is that the same direction is still conserved in all bodies together that are assumed as interacting, in whatever way they come into collision. If this rule had been known to M. Descartes, he would have taken the direction of bodies to be as independent of the soul as their force; and I believe that that would have led direct [*sic*] to the Hypothesis of Pre-established Harmony, whither these same rules have led me. For apart from the fact that the physical influence of one of these substances on the other is inexplicable, I recognized that without a complete derangement of the laws of Nature the soul could not act physically upon the body (Leibniz 1985, p. 156).

To paraphrase, Descartes’s volume × speed quantity is not conserved, but momentum (mass times velocity, including direction) is conserved, so the soul’s deflecting matter while leaving its speed unchanged violates the newer notion of conservation. Being a rationalist, Leibniz had high aesthetic standards for physics and metaphysics. While co-inventing calculus, Leibniz did not inherit (as we do) an expectation that physical laws would come as differential equations that generically cannot be solved exactly. Hence what he counted as a “complete derangement” might not be nearly so disturbing nowadays. From Leibniz till the mid-18th century, this issue was intimately bound up with the *vis viva* controversy, which was roughly a disagreement about whether there ought to be a conservation of energy law. (Laudan 1968; Smith 2006). The debate about conservation and the soul engaged Knutzen, Crusius, Kant, Maxwell, Helmholtz, Broad, and others.

This concern continues to influence discussions in the contemporary philosophy of mind, often with an expectation of a quick and decisive victory over dualism. Many contemporary philosophers of mind invoke the conservation of energy against interactionist dualism (Bunge 1980, p. 17; Morowitz 1987; Pollock 1989, p. 19; Flanagan 1991, p. 21; Dennett 1991, pp. 34, 35; Fodor 1998, p. 64; McGinn 1999, p. 92; van Inwagen 2002, p. 196; Searle 2004, p. 42; Lycan 2011; Westphal 2016, pp. 41-44; Schweizer 2019) (and more in lists in ((Montero 2006; Collins 2008; Gibb 2010))). Dennett expresses the objection as follows:

It is this principle of the conservation of energy that accounts for the physical impossibility of “perpetual motion machines,” and the same principle is apparently violated by dualism. This confrontation between quite standard physics and dualism has been endlessly discussed since Descartes’s own day, and is widely regarded as the inescapable and fatal flaw of dualism (Dennett 1991, p. 35).

Even dualist E. J. Lowe thought that conservation laws might be problematic for interactionist dualism. Lowe proposed several ways out, one of which (though not his favorite) has merit (Lowe 1992, 1996, pp. 56-61; Lowe 2003, pp. 138, 139). This 20th-21st century debate of course continues a discussion with important 18th and 19th century contributions (Watkins 1995, 1998; van Strien 2015; Heidelberger 2003); a future work will survey the history normatively in more detail (Pitts 2019a).

Bunge has feared that physics and much else would collapse if dualism were true.

*Dualism violates conservation of energy.* If immaterial mind could move matter, then it would create energy; and if matter were to act on immaterial mind, then energy would disappear. In either case energy—a property of all—and only concrete things would fail to be conserved. And so physics, chemistry, biology, and economics would collapse. Faced with a choice between these “hard” sciences and primitive superstition, we opt for the former (Bunge 1980, p. 17).

Why body-to-soul causation should make energy disappear is quite unclear (Rosenthal 1998; Shaffer 1968, p. 66; Russell 1927, p. 157). Immaterial souls (or non-redundantly causally efficacious dualist mental properties) are not physical, after all, so why must affecting them take energy? Soul-to-body causation, on the other hand, certainly *should* violate the conservation laws; this all but follows from Noether’s first theorem, which derives conservation laws from symmetries and which has a converse. However, it is doubtful that even a Sith lord or Sauron could make physics, chemistry, biology and economics collapse with mental force; why else would they bother with armies with blasters or swords? The property of gentle failure (below) addresses Bunge’s concerns. In any case, if perchance it were to be found empirically that immaterial souls really do act on bodies, perhaps 21st century neuroscience might encounter some bumps, but no other discipline would be much affected. Physicists and engineers routinely study systems with external influences, such as the driven harmonic oscillator (Marion and Thornton 1988), without encountering any generic pathology.

As various authors have noted, non-epiphenomenalist forms of property dualism suffer from analogous worries about how the mental can affect the physical (Crane 2001, pp. 40, 43, 50; Searle 2004, pp. 44-46; Zimmerman 2007; Lycan 2013). Nondualist views have quite different problems with mental causation (Gibb et al. 2013). Because having some problem with mental causation is common, and property dualism (if not epiphenomenalist) can have the same kind of problem that interactionist substance dualism has long had, the status of the energy conservation objection is of more than historical interest (whether or not one takes substance dualism to be an option today). For brevity and in view of the widespread traditional opinion that interactionism is the most plausible form of dualism, interactionist dualism (whether of substance or property) will henceforth be called “dualism”.

The plan of this paper is as follows. I will survey extant views on this longstanding objection. A formalization will be attempted of the argument that objectors seem to envisage. A survey a variety of features of conservation laws that are frequently neglected in the philosophy of mind follows. (Typically the physics employed in the conservation discussion is that covered in high school chemistry and corresponds roughly the physics of the 1860s or earlier.) Especially but not only because of conditionality, it emerges that this longstanding (1690s-2010s) objection *begs the question*. This claim has sometimes been made, but rarely in a way that has seemed compelling. Then I review the best extant work on the topic (Averill and Keating 1981). Addressing the locality of conservation requires briefly sketching the history of views about the relations between minds and space. Then conditionality is studied in more mathematical detail, followed by locality in more detail. (Readers less interested in the mathematics can skim these sections.)

I conclude by calling attention to two more serious difficulties, empirical neuroscience and a novel energy conservation objection from General Relativity (Pitts 2019b). The latter does not obviously beg the question in the fashion that the traditional objection from Leibniz does—though it could have more subtle flaws. These two issues, not the traditional but unsuccessful Leibnizian objection, should be discussed in the future.

## Extant Dualist Responses

Dualists have made a variety of responses to the conservation law objection, some clearly incorrect, some incorrect but interesting, and some correct but quite incomplete. The plausibilities ascribed to the various responses within the philosophy of mind literature has little correlation to their physical plausibilities.

### Incorrect Dualist Responses

One incorrect dualist response to the conservation law objection has been to deny that any violation of conservation occurs (Broad 1937, pp. 106-109; Shaffer 1968, pp. 66, 67; Beloff 2002; Dilley 2004; Meixner 2008; Gibb^2010^; White 2017). Even oversimplifying the physics, such as by thinking in terms of particles and global conservation rather than continua and local conservation, one still runs afoul of Noether’s first theorem and its converse as applied to time translation invariance (the temporal uniformity of nature), as will appear below. One also has the less famous but equally severe problem of momentum conservation (Cornman 1978). While the conservation of momentum issue apparently took 40 years to reappear in response to Broad, the problem in principle should have been clear using the physics of the 1830s, when the connection between momentum conservation and the homogeneity of space was clearly stated in relation to variational principles (Hamilton 1834; Jacobi 1996; Marion and Thornton 1988), not to mention Leibniz’s invocation of momentum conservation (Leibniz 1985, p. 156), which did not require a variational formulation. One sees the value of talking not about closed systems, but about symmetries (implying conservation laws) or violated/broken symmetries (not implying conservation laws): it is difficult to say whether a physical system potentially subject to mental influences is “closed” (that being a distinction not intended for the philosophy of mind), but it is perfectly clear that such a physical system lacks the symmetries of time- and space-translation invariance in the regions of mental influence. Cornman’s attention to contingent details is not needed; the mere fact (if it is a fact) that my mind acts on my body and not on Mars implies that mental causation (on the view in question) violates momentum conservation.

A related incorrect dualist response is teleportation, that is, nonlocal compensation so that energy that disappears in one place and appears elsewhere (simultaneously?) (Ducasse 1960). This answer is a bad idea whether the compensation is thought to happen simultaneously or at some retarded time (taking into account the speed of light), because this response neglects the fact that in modern physics, matter is continuous and conservation laws are local, as will be discussed more below. One reason to insist on local conservation is the relativity of simultaneity. That is by no means the only reason, however. Because modern physics consists of local field theories, conservation laws are local (Goldstein 1980, pp. 550, 556; Griffiths 1989, p. 4; Lange 2002, chapter 5). Even if perchance relativity someday falls in favor of absolute simultaneity—a possibility less extravagant after 2009 than one might have thought (Hořava 2009) (cited over 1900 times thus far!)—one should still expect local conservation laws, because matter is continuous and acts locally through the propagation of waves. Because conservation laws in real physics are local, nonlocal conservation aims to rectify a violation with what is, in fact, another violation.

Another incorrect response is the idea that the soul might be able to alter the value of physical constants (even only locally and slightly) (Lowe 1992). Even if the soul could do such a thing (which seems even less natural from a physical standpoint than the mental force discussed below), such a proposal would violate conservation laws just as badly as would direct mental action on the brain, as will become clearer in light of the converse first Noether theorem. Spatio-temporally varying ‘constants’ would remove the time-translation and space-translation symmetries that entail (and are entailed by) the local conservation of energy and momentum (Cucu and Pitts 2019).

Another incorrect response is to hold that energy is conserved through dualist interactions because physical energy is converted into mental energy (Hart 1994). Unless one is prepared to ascribe mathematical properties to mind, talk of mental energy is a category mistake or an equivocation between two basically unrelated concepts. If one is willing to ascribe mathematical properties to the mind itself along the lines of classical field theory (which seems absurd), then talk of mental energy requires exhibiting the mathematics defining the canonical energy-momentum tensor (on which more below).

Conservation laws are also invoked as an objection to claimed parapsychological phenomena, such as the table levitation of certain late 19th-early 20th century mediums. (Griffin provides many references (2000, chapter 7).) Braude, who accepts such phenomena and reports witnessing a table tilting in graduate school, argues that such phenomena are compatible with energy conservation due to cold breezes and changes in the weight of the medium (Braude 1986, 1987; Braude et al. 2017). This proposal has the virtue of addressing both energy and momentum conservation, it would seem. It may combine several of the above supposed compensation mechanisms. In any case it is clear that conservation, being not just global but primarily local, and being related to symmetries by Noether’s first theorem and converse, is too demanding to be compatible with any such phenomena.

### More Interesting Dualist Responses

Dualists have also made some interesting responses to the conservation law objection, some of them correct but incomplete, others incorrect but instructive. One interesting dualist response is the claim that conservation fails but that is no objection because the law is conditional on the lack of outside influence; let us call this the “conditionality” response. Garber and Lowe have offered this response as a reasonable option on Descartes’s behalf (Garber 1983; Lowe 1992); Rodrigues and Lycan offer it as an option for dualists (Rodrigues 2014; Lycan 2018). Meixner has mentioned conditionality sympathetically without relying upon it because he considers only global conservation laws (Meixner 2008). Others have endorsed it, including Knutzen and Crusius long ago (Watkins 1998, 1995) (influencing the young Kant) and some more recent authors (Ducasse 1960; Averill and Keating 1981; Larmer 1986; Plantinga 2007). This response is correct. It also applies to momentum conservation. It needs further development, however, which will be given below.

A second interesting dualist response is the claim that conservation laws already fail in General Relativity even apart from dualism (Mohrhoff 1997; Collins 2008; 2011).^1^ If conservation already fails given General Relativity, then plausibly there is no conservation remaining for dualism to spoil, so the usual objection is eliminated. This is an impressive aikido-like move, rhetorically, and shows much better grasp of the physics than usual. Unfortunately the truth is the reverse: General Relativity makes Cartesian mental causation *harder*, not easier (see also Pitts 2019b). One might criticize the Mohrhoff-Collins idea by taking the formal (“pseudotensor”) conservation laws and gravitational energy in General Relativity seriously, one might say a realist interpretation (Pitts 2010). Surprisingly to those outside the field of General Relativity, it is unusual to take the formal conservation laws and gravitational energy seriously in General Relativity. The Mohrhoff-Collins proposal takes a widely shared idea in the General Relativity literature and derives a startling conclusion. In my experience, mentioning this argument at physics conferences tempts physicists to apply *modus tollens* (as I do) rather than *modus ponens*; perhaps this is a reason to take gravitational energy more seriously? The status of gravitational energy and its conservation is relevant to conserved quantity theories of causation (Fair 1979; Rueger 1998; Dowe 2000; Curiel 2000). Taking gravitational energy seriously, as I do, would be an asset for conserved quantity theories of causation; mine is, however, thus far a minority view.

Fortunately for the issue of mental causation, one can go beyond interpretive stances and perform uncontentious novel *calculations* using the generalized Bianchi identities. In the simplest case, when the nonphysical mental influence is a scalar field (a single number at each point, the same in all coordinate systems), it follows mathematically that influence must be spatio-temporally constant. That is absurd for a human (finite) mind unless the influence is simply zero: there is no influence. Thus General Relativity makes nonphysical mental causation *harder*, not easier, a rigorous conclusion that is *entirely independent of interpretive stances about gravitational energy, pseudotensors and local conservation laws*. Realism about gravitational energy has the correct heuristic force, which perhaps counts in favor of the view. But one by no means needs to sympathize with such realism to accept the mathematical argument. This issue will be discussed elsewhere (Pitts 2019b). *This new objection to dualism does not beg the question in the fashion that the traditional objection from Leibniz till today does.* Whether the simplest case is representative is another matter.

While common in the philosophy of mind, the traditional (not general relativistic) argument from conservation against dualism is rarely made by experts in physics even when they discuss the mind. Thus noted philosopher of physics Jeremy Butterfield writes:

… [A]traditional argument against interactionism is flawed, because of this false picture of physics.… The idea is that any causal interaction between mind and matter would violate the principle of the conservation of energy.… But, says the argument, physics tells us that energy is conserved in the sense that the energy of an isolated system is constant, neither increasing nor decreasing.… And there is no evidence of such energy gains or losses in brains. So much the worse, it seems, for interactionism. (Though traditional, the argument is still current; for example, Dennett endorses it (1991, pp. 34-35).) This argument is flawed, for two reasons. The first reason is obvious: who knows how small, or in some other way hard to measure, these energy gains or losses in brains might be? Agreed, this reason is weak: clearly, the onus is on the interactionist to argue that they could be small, and indeed are likely to be small. But the second reason is more interesting, and returns us to the danger of assuming that physics is cumulative. Namely: the principle of the conservation of energy is not sacrosanct. … [A]lthough no violations have been established hitherto, it has been seriously questioned on several occasions. It was questioned twice at the inception of quantum theory…. And furthermore, it is not obeyed by a current proposal … for solving quantum theory’s measurement problem. In short: physicalists need to be wary of bad reasons to think physicalism is true, arising from naivety about physics. (Butterfield 1997)

This quotation has several noteworthy features. First, Butterfield does not even bother to entertain the common “brain-free” argument (which claims that there is a problem quite apart from looking at the brain), presumably because it is obviously circular. Rather he moves straight to a more promising *a posteriori* empirical version: “there is no evidence of such energy gains or losses in brains.” Part of this paper’s aim will be to explain what Butterfield understood, that the argument only starts to have force once one looks at the brain. Second, as also noticed in part by Papineau (2002, p. 45), philosophers of physics and physicists have a quite different view of conservation laws from philosophers of mind. Many philosophers of mind regard conservation laws as categorical results requiring submission from metaphysics and the philosophy of mind. By contrast Butterfield and physicists know where conservation laws come from (namely symmetries), naturally think quantitatively (including approximations) rather than thinking of conservation as simply holding or failing, and do not necessarily find the prospect of small violations frightening. Third, quantum physics might provide a reason to accept small occasional violations of conservation laws and so might be a resource for dualists. Indeed quantum mechanics has been suggested as ‘leaving more room’ for mental causation by a number of authors including Arthur Eddington, Eugene Wigner, Henry Margenau, Karl Popper and John Eccles, Henry Stapp, Roger Penrose, Walter Freeman and Giuseppe Vitiello, Hans Halvorson and Adrian Kent. (Some of these authors hold that a quantum account has the virtue of *avoiding* non-conservation, as opposed to making non-conservation permissible; partly at issue is how one formulates conservation laws *vis-a-vis* collapse of the wave function, if such occurs).^2^

However that might be, I will ignore quantum physics, leaving that potential dualist resource on the table. Clearly quantum mechanics makes matters no worse for the dualist, whether or not it makes them better. My task will be to make clear what is known theoretically about conservation laws in the most conservation-friendly realm of theoretical physics, namely, classical field theory. If quantum field theory is our ultimate physics, classical field theory is our penultimate physics, the starting point from which the wonder of quantization is performed by adding the symbols “$\hat{\mkern6mu}$” to equations to turn functions into operators. When the common objection to dualism from conservation laws is seen to fail even in its most congenial environment, *a fortiori* it fails in quantum field theory. If one wants to give a plausible positive story about soul-body interaction (if one is possible), certainly quantum physics will be required. But if one only aspires (as I do) to evaluate the relevance of conservation laws to the possibility of soul-brain interaction, then one can ignore quantum physics because classical field theory gives conservation its best shot, and still no interesting objection arises. Feigl, who played an important role in 20th century philosophy of mind, might have understood that there was no interesting energy conservation objection. While discussing another question, he mentions the conditional: *If* the law of the conservation of energy holds, then a *perpetuum mobile* (of the “first kind”) is thereby logically excluded. But, of course, the energy law has only empirical validity and might some day be refuted by cogent empirical evidence (Feigl 1958, p. 472).

General Relativity, on the other hand, is a special classical field theory that poses a novel difficulty for mind-to-body causation, as appeared above, *pace* claims that General Relativity provides a new escape from the old difficulty. Thus it could turn out that quantum considerations matter once one considers General Relativity.

## The Brain-Free Argument

The conservation of energy is one of the most memorable things that one learns in secondary school physical science courses such as chemistry and physics. It seems entirely appropriate that one employ this scientific knowledge when doing metaphysics and the philosophy of mind. Thus metaphysicians and philosophers of mind, one might reason, should impose the conservation of energy as a constraint on acceptable views. Dualism is often suspected of violating the conservation of energy. One can thus appeal to *modus tollens*:

1. Dualism implies that energy is not conserved.
2. Energy is conserved. (from Science)
3. Therefore, dualism is false. (by *modus tollens*)

This is a valid argument with plausible-looking premises. If the argument is sound, then dualism is false. This type of reasoning seems to be frequent. One might almost call this attempted *reductio* “a priori” because no investigation of the *brain* is required, though of course premise 2 is not wholly a priori. I will call it a “brain-free” argument.

Ladyman et al. have warned us, however, that not everything aiming to be naturalistic metaphysics (in the sense of respecting science) is altogether successful.

We might thus say that whereas naturalistic metaphysics ought to be a branch of the philosophy of science, much metaphysics that pays lipservice to naturalism is really philosophy of A-level chemistry (Ladyman et al.^2007^, p. 24).

This anti-dualist argument is naturalistic in the sense of opposing spirits, but its naturalistic character in the science-respecting sense is not so evident. Russell said some time ago that “philosophers … are too apt to take their views on science from each other, not from science.” (Russell 1913)

Some dualists have accepted premise 2 and denied premise 1 in an attempt to respect science. Unfortunately the opposite is achieved, at least in relation to physics (as opposed, perhaps, to neuroscience). Premise 1 is true in light of the Noether biconditional relationship between symmetries and conservation laws, as will appear below. In the context of a debate on mental causation, premise 2 is question-begging unless one has found it to be true partly through neuroscience. Without investigation of the brain, positive instances of energy conservation are (according to the dualist) from a fatally biased sample. Given the well known collection of non-frivolous a priori arguments for dualism, requiring further evidence for conservation in brains is not like an unprincipled request for requiring further evidence for conservation in my toothbrush, or on Vanuatu, or whatever.

Premise 2 is often supposed to be empirically well confirmed. Doubtless a great many positive instances could be mentioned, many of them involving steam engines; most of them have nothing to do with contexts where spiritual influence was antecedently plausible to anyone. A logical question arises about the one-sidedness of the evidence for conservation laws, according to Larmer. “Faced with reports of miracles, the occurrence of which would constitute evidence that energy can be created or destroyed, it begs the question to dismiss such reports as antecedently improbable on the grounds that they imply the falsity of the claim that energy can neither be created nor destroyed.” (Larmer 2014) Probably everyone rejects at least some miracle reports and hence filters the evidence in light of broader considerations; the typical *orthodox* Protestant viewpoint denied that miracles have occurred for over 1500 years (Warfield 1918; Craig 1985). Whether or not one considers it more reasonable to filter out the remaining miracle reports as well, the fact remains that one is filtering the evidence in light of larger considerations. Thus the seemingly uncontentious claim that the conservation of energy is empirically well confirmed (*always and everywhere*) hides a worldview-laden conclusion that one has managed to filter out (to one’s own satisfaction) whatever counter-evidence one has encountered. Evidently, more directly relevant evidence for energy *non*-conservation pertaining to brains is even scarcer.

## Overview of Conservation Laws

This section provides an overview of features of conservation laws in modern physics. Some of these features require a more detailed discussion, which will be given in later sections. Lange (2002, chapter 5) gives a useful discussion of the conservation of energy and momentum and their relations to spatial distribution, but this material is still little known in the philosophy of mind. When the true logical form of conservation laws is recognized, the failure of both the Leibnizian objection and several dualist responses becomes evident.

### Locality

First, conservation is fundamentally *local*: energy conservation holds not primarily for the whole universe, but in every place separately. Indeed the global conservation law can fail to make sense in cosmology, if the relevant integrals diverge (Peebles 1993, p. 139), whereas the local conservation laws remain meaningful. A few works in the philosophy of mind (by dualists, as it happens) have noticed that conservation laws are local (Hart 1988, p. 64; Plantinga 2007). But many dualists have suggested that energy conservation still holds if the mind affects only the *distribution* of energy, not the total amount (Broad^1937^, p. 109; Dilley 2004; Meixner 2008; Gibb 2010). This dualist response assumes a (merely) global conservation of energy: for all times *t*, the energy *E*(*t*) for the whole universe is the same, then one can write merely *E*. Campbell, no friend of dualism, generously grants this possibility (Campbell 1984, chapter 2). But that is a mistake. Time differentiation gives the more generalizable differential version

1 $$ \left(\forall t\right)\frac{dE}{dt}=0. $$

But that is not the form that the conservation laws have taken since the late 19th century, with Hertz’s discovery of “electric waves” (electromagnetic radiation) as predicted by Maxwell’s theory and with the concomitant propagation of energy at the speed of light (Poynting 1884) (not instantaneous action at a distance, though it took some time to extend the result to gravity). One now understands matter to be continuous, represented by various mathematical fields, which are functions of time *and space.* (If one takes account of quantum mechanics, one gets quantum field theory (Kaku 1993; Peskin and Schroeder 1995), but it has many conceptual and technical difficulties (Duncan 2012).) One can then define the density of energy *ρ*(*t*,**x**) for every space-time location (*t*,**x**). (In vector calculus, **x** is the spatial position. Below a transition will be made to the more expressively adequate component notation.) In the same way one can define the energy current density **J**(*t*,**x**). The conservation of energy then takes the form

2 $$ \left(\forall t\right)\left(\forall \mathbf{x}\right)\left[\frac{\mathrm{\partial \uprho}\left(t,\mathbf{x}\right)}{\mathrm{\partial t}}+\nabla \cdot \mathbf{J}\left(t,\mathbf{x}\right)=0\right], $$

where

3 $$ \nabla \cdot \mathbf{J}=\frac{\partial {J}_x}{\mathrm{\partial x}}+\frac{\partial {J}_y}{\mathrm{\partial y}}+\frac{\partial {J}_z}{\mathrm{\partial z}} $$

is the “divergence” of the current density vector field **J** (with magnitude and direction at each point), a measure of how fast energy spews out of the place. The quantification over spatio-temporal locations is important when one considers negation: just as the negation of *A*&*B* is ¬*A* ∨¬*B*, so the negation of this universally quantified conservation law is simply that *there exists* a space-time location where $\frac{\mathrm{\partial \uprho }}{\mathrm{\partial t}}+\nabla \cdot \mathbf{J}\ne 0,$ i.e., at which energy is not conserved. Energy might still be conserved almost everywhere and almost always. Non-conservation might also be small even where it occurs. Everything said here for energy also applies for momentum in three directions and for angular momentum in three directions as well. The conservation of mass is of course no longer a valid conservation law in general, but mass-energy plays its part in the conservation law for energy.

### Conditionality

Second, whereas many philosophers believe energy conservation to be a *categorical* result of physics (e.g., Fales 2010, p. 13), a few authors writing on the philosophy of mind have rightly asserted that energy conservation in theoretical physics is *conditional* upon the absence of external influences (Ducasse 1960, p. 89; Averill and Keating 1981; Larmer 1986; Plantinga 2007; Moreland and Rae 2000, pp. 107, 108).^3^ External influences that vary with time lead to the non-conservation of energy; external influences varying with location lead to the non-conservation of momentum (Lagrange 1997; Hamilton 1834; Jacobi 1996; Marion and Thornton 1988; Goldstein 1980). Dualism claims that immaterial souls affect bodies; but souls are not (present? and) active in the same way everywhere and always, so any causal influence from the soul on the body will vary with time and place, leading to the non-conservation of energy and momentum where and when they act (and only there and then). If my soul (supposing that I have one) causes my arm to rise, the mental force is exerted on Earth in the 21st century, not on Mars 100 years ago. Here it is worthwhile to recall the difference between 17th-18th century Cartesian mechanical philosophy and the vitalistic doctrines of 18th-19th century *Naturphilosophie* and Romanticism (for example, Strawson 2008). The latter insisted that life was different from ordinary matter, whereas the former claimed that only mind differed fundamentally from matter, while life was merely a complex mechanical arrangement of matter. While various 19th century physiological experiments counted heavily against vitalism, they have little or no force against a neo-Cartesian view in which souls act on brains; that requires looking at the brain. That energy is conserved in the stomach and the elbows would be no surprise to Cartesian mechanical philosophers. Graeco-Roman medicine already arrived at the conclusion of the centrality of the brain to thought (Solmsen 1961). Lewis found Jackson’s knowledge argument against materialism powerful, but then offered a dilemma: either epiphenomenalism is true and the knowledge argument seems absurd because the qualia make no difference, not even helping to cause utterances about qualia, or the qualia make a difference and one is betting “against the truth physics”, which seems “rash” (Lewis 1999) On the contrary, one is betting against naturalism and might be betting against neuroscience, and that might be rash, but one is not betting against physics; physicists might defend Lewis’s claim if they are materialists but would deny it if they are dualists. Historically Euler comes to mind as a vigorous proponent of interactionist dualism, critic of Leibniz-Wolff pre-established harmony, and defender of Christianity (Euler 1840, parts of Letters 79-115 (Volume I) and of Letters 1-17 (Volume II)) (Breidert 2007), not to mention the dominant figure in 18th century physics, inventing much of continuum mechanics with its local conservation laws and leaving his name on the Euler-Lagrange equations.^4^ One might, e.g., take the view that laws of nature express natural tendencies but fail to specify whether there exist immaterial substances and what those might cause (von Wachter 2006).

### Noether Converse: Conservation Implies Symmetry

While the conditional nature of conservation laws is sometimes recognized, there is a little-known converse theorem that strengthens the point. Noether synthesized and generalized some extant results (e.g., Herglotz 1911; Mie 1913; Born 1914; Pitts^2016^) regarding conservation laws in Lagrangian theories of continua/fields in her famous first theorem. But she also proved a converse: one can infer from conservation laws to symmetries (Noether 1918; Brading 2001; Brown and Holland 2004; Kosmann-Schwarzbach 2011; Romero-Maltrana 2015). By contraposition,

Lemma: the falsity of symmetries implies the falsity of conservation laws.

Thus it is evident by inspection that the soul’s acting causally on the brain will tend to violate conservation laws.

Aristotle understood that scientific demonstrations should have premises known better than and prior to the conclusions (Smith 2015). The difficulty for the anti-dualist objector from conservation laws is that, given Noether’s theorem and its converse, *the distance between premise and conclusion has all but disappeared*. Hence the circularity complaint arises. What formerly looked like an argument against dualism now looks like a *mere incredulous stare*: the problem with souls pushing on bodies is that then souls push on bodies. *Of course* conservation laws fail, given dualism; that follows from the converse Noether theorem from conservation laws to symmetries. But if one finds forceful the sorts of a priori arguments that dualists use (as even some non-dualists admit (Strawson 2008; Nagel 2012)), one ought not to be dissuaded by an objection that one is thus committed to an immaterial spatio-temporally varying influence on matter. That is a restatement of the dualist position followed by an unmotivated appeal to *modus tollens*, when *modus ponens* was also available to the dualist. An appropriate response by a physically informed dualist to the usual energy conservation objection would be “yeah, so what? Have you looked at the brain closely enough?” Given that some dualists, trying to respect science, have striven to keep dualism consistent with conservation laws, Noether’s converse will have force against those dualist views. But successfully respecting physics requires an adequate understanding of the physics, not merely a disposition to submit to what one thinks physics teaches. Ladyman et al. have distinguished between domesticated and genuine physics (Ladyman et al. 2007, p. 24). Ironically, those philosophers who are willing to see conservation fail (Ducasse 1960, p. 89) (Averill and Keating 1981; Larmer 1986; Plantinga 2007) (some of whom are religious a priori metaphysicians) have a more accurate view of the theoretical physics and hence a more naturalistic metaphysics in one sense. (That is not necessarily the same as a more accurate view of neuroscience, or of science in general, if there is any such thing). Apparently the “quite standard physics” of conservation laws is not what it seems from more elementary presentations. If there is a fatal flaw for dualism in here somewhere, it would be helpful to see an argument that isn’t obviously circular in light of 20th century physics and that takes into account the fact that dualism itself is motivated by plausible arguments.

The Noether converse inference from conservation to symmetry excludes an idea recently proposed.

In short, the dualist might argue that the postulation of non-physical, mental forces is not at all in tension with acceptance of CoE, because the conservative nature of the basic physical forces we now know of gives us ample reason to think that any non-physical, mental forces there are will likewise obey CoE.

… [T]his is, I think, the right thing for the dualist to say…. (White 2017)

White needs an account of the mathematical details of a scheme to preserve conservation laws without forcing the alleged mental influence on the physical to be the same everywhere and always, but Noether’s converse first theorem stands in the way.

### From Conservation to a Form of Causal Closure; But So What?

Regarding whether the conservation of energy provides an argument for causal closure of the physical, Broad and Papineau bear mention. Broad claimed that the conservation of energy was “absolutely irrelevant” in trying to criticize two-way mind-body interactionism (Broad 1937, p. 104). Certain physiological experiments indicating energy conservation in the body were “completely irrelevant” (Broad^1937^, p. 106). He went on:

Thus the argument from energy has no tendency to disprove Two-sided Interaction. It has gained a spurious authority from the august name of the Conservation of Energy. But this impressive principle proves to have nothing to do with the case (Broad 1937, p. 109).

Broad’s grasp of physics (1919) was deeper than one might gather from *The Mind and its Place in Nature*, though not deep enough in this case. Papineau has a nuanced discussion which sees the conservation of energy as a contributing factor to the eventual widespread acceptance of the causal completeness of physics (Papineau 2000). Fortunately he places considerable stress on empirical considerations from physiology (pp. 202, 203).

The converse first Noether theorem (Noether 1918; Kosmann-Schwarzbach 2011) is crucial in this context in two respects. On the one hand, the converse first Noether theorem says that conservation laws imply symmetries. Symmetries all but imply causal closure, because any mental forces should vary with time and place, as human minds obviously do.^5^ The assumption of the conservation of energy and momentum at every point in space (local conservation laws) at every moment of time implies (in the context of classical field theory and the principle of least action) that the laws of physics are invariant under rigid time- and space translations: the temporal and spatial uniformity of nature. Hence any mental influence Ψ(*t*,*x*,*y*,*z*) must be the same everywhere and always (Ψ = *constant*). But surely my willing to raise my arm on Earth in 2018 does not have a uniform influence everywhere and throughout the whole history and future of the universe. Thus Ψ = 0 is the obvious boundary condition: the ostensible mental influence disappears, yielding an intuitive version of causal closure: the mind doesn’t actually have any effect on the world. Energy and momentum conservation, rightly understood, all but entail an intuitive version of causal closure and the closure of the gap is obvious. Papineau generously entertains (as a serious option historically) the possibility of mental forces that respect the conservation laws (Papineau 2000, 2009). Papineau might well be correct about what Victorian thinkers generally thought; the wondrous example of Stewart and Tait’s interpreting divine interposition of energy in our world as in fact a transfer of energy from an unseen realm, with total energy conserved, comes to mind (Stewart and Tait 1890; Heimann 1972). But in light of the principle of least action (Born 1914; Noether 1918), it should be clear that trying to uphold energy conservation while admitting mental forces conflicts with the Noether relation between symmetries and conservation laws. Given the simpler versions of the inference from symmetry to conservation already available long since in their day (Lagrange 1997; Hamilton 1834; Jacobi 1996), that isn’t even clearly hindsight.

On the other hand, the assumption of energy (and momentum) conservation—that is, the assumption of conservation *everywhere and always*—is a much stronger and more conjectural claim than one might have thought. For example, the success of energy (and momentum) conservation in physics and in biology (including the absence of vital forces) arguably has hardly tendency to undermine Cartesian mental forces; Descartes himself would have predicted such success. Arguably more relevant is the apparent (empirical?) progress in reducing the number of plausible exceptions to physical laws (such as miracles) over the last 500 years. The empirical sword could be double-edged, however (Broad^1937^, ch. 12). The logic is not luminous, either (Earman 2000). So while conservation all but entails causal closure of the physical, conservation is far less certain that one might have thought, so no decent argument for the causal closure of the physical results. What the Leibnizian critic of broadly Cartesian views needs is some reason to think that conservation laws hold even if minds are assumed to act on bodies, but such reasons are difficult to find without begging the question, at least if one is not looking at the brain.

### Gentle Failure

Another insufficiently recognized feature of conservation laws is a robustness property that one might call gentle failure: energy conservation fails gently if it fails at all, so a modest localized external influence causes no universal catastrophe overthrowing all science everywhere, *pace* Bunge. Some authors who appreciate the conditional nature of conservation laws, by falling silent after asserting the conditionality, seem to suggest that where conservation does not hold, there is just nothing to say. That silence could be worrisome. If my soul can create enough energy to tell my brain to make my finger move, can it create an airplane on a runway? Can I blow up with world with pure thought? Inductive inference and science generally could be imperiled, much as Bunge feared. Perhaps physics experiments would be overthrown by energy leaking in from massive nonconservation in the experimenters’ brains? Fortunately one can distinguish degrees of nonconservation, not only in sense of the spatio-temporal region where violation occurs, but also in the magnitude of nonconservation at any given point. As engineers know, where a conservation law fails due to a source or sink, one has instead a balance law relating the density and flux of the quantity in question to that source or sink:

4 $$ \frac{\mathrm{\partial \uprho }}{\mathrm{\partial t}}+\nabla \cdot \mathbf{J}= source $$

(Misner et al. 1973, p. 567; Müller and Ruggeri 1998, pp. 21-26). If the non-conservation is everywhere and always either zero or *sufficiently small* (especially if the places where it is non-zero are rare or relatively inaccessible), there is little threat to the uses made of and apparent empirical knowledge expressed by conservation laws (Butterfield 1997). Thus occasional small failure of the conservation laws would not threaten chemistry, biology or economics, nor even most of physics.

## Averill and Keating’s Neglected Work

After this survey of aspects of conservation laws generally neglected in the philosophy of mind literature, it is appropriate to revisit the best extant work known to me. Averill and Keating’s treatment (1981) has been the most successful in interacting with real theoretical physics, drawing upon a standard graduate-level mechanics textbook, (the first edition of) Herbert Goldstein’s *Classical Mechanics*, and often getting things right. It is therefore worthwhile to point out both how much they got right (much of which is still widely denied) and how much remained to do beyond their treatment. Commendably, they avoid talk of closed systems in favor of talk of the presence or absence of external (especially mental) force.

Averill and Keating rightly talk about mental force but not mental energy. Invocations of mental energy (Hart 1988; Meixner 2004; Hart and Yagisawa 2007; White 2017) make little contact with the mathematical physics of conservation laws. To exert a force simply requires coupling to a physical system, but having energy involves making *its own contribution to definition of energy via* a term along the lines of $\frac{\mathrm{\partial L}}{\mathrm{\partial \dot{z}}}\dot{z}$ for some quantity *z*; presumably nonphysical minds or mental properties do not do that. (An analogy might help at least the many who find affinities between dualism and theism: traditional theists do not regard God as *having* or *transferring* energy (Fales 2010, p. 26), even if God can impart energy.) Regardless, one should expect conservation to fail because the laws of physics vary with time and place on account of the soul’s influence. Thus there is no tension between Averill and Keating’s rejection of mental energy and their embrace of mental force. An example below will make these matters more explicit.

Most importantly, Averill and Keating recognize the conditionality of the conservation of momentum and energy and thus point out that since dualism contradicts the antecedent (no external force), dualism’s contradicting the consequent (the conservation of energy and momentum) cannot be refuted merely by talking about supposed but overly strong ‘laws’ of conservation. No true conservation ‘law’ is violated even if conservation fails.

But much is left undone. Averill and Keating do not frame conservation in terms of symmetries (energy due to time translation symmetry, momentum due to space translation symmetry), early 19th century results (if not earlier) extended by Noether in the 1910s. They are (like many experts in physics!) not aware of Noether’s converse results, results which make all the clearer the question-begging nature of the objection to interaction from conservation laws because the premise and conclusion nearly coincide. Finally, Averill and Keating do not recognize the locality of conservation laws, a topic discussed by Goldstein in the concluding chapter, which covers continuous systems and fields.

With this brief mention of various under-recognized features of conservation laws completed, now a more thorough discussion of a few of these properties is in order. While gentle failure and the Noether converse seem not to need further discussion, the conditionality issue, the distinction between mental energy (implausible) and mental force (less implausible), and the locality of conservation laws need more detailed discussion. The crucial relevance of General Relativity will be discussed elsewhere (Pitts 2019b).

## Example; Mental Force Without Mental Energy

At this point an example might be useful. One of the simplest examples, and yet one useful for present purposes, is that of a particle able to move only vertically in a constant gravitational field, a typical approximation used near the surface of the Earth. Let *z*(*t*) be the height of the particle at time *t*, $\dot{z}(t)$ be its velocity, *m* (a constant) be its mass, and *g* (a constant) be the acceleration due to gravity. The Lagrangian function (a.k.a. the “Lagrangian,” the kinetic energy minus the potential energy) is

5 $$ L=\frac{1}{2}m{\dot{z}}^2- mgz. $$

The Euler-Lagrange equation is

6 $$ {\displaystyle \begin{array}{rcl}\frac{\mathrm{\partial L}}{\mathrm{\partial z}}-\frac{d}{dt}\frac{\mathrm{\partial L}}{\mathrm{\partial \dot{z}}}& =& 0\\ {}& =& - mg-\frac{d}{dt}\left(\frac{1}{2}m\cdot 2\dot{z}\right)\\ {}& =& - mg-m\ddot{z}=0.\end{array}} $$

This is Newton’s second law in the form *F* − *ma* = 0. More relevant for current purposes is the fact that, in this case, one can read off the violation of momentum conservation by inspection. The momentum of the particle is $\frac{\mathrm{\partial L}}{\mathrm{\partial \dot{z}}}= m\dot{z}$. The Euler-Lagrange equation specifies the rate of change (time derivative) of the momentum $\frac{d}{dt}\frac{\mathrm{\partial L}}{\mathrm{\partial \dot{z}}}$ in terms of the force $-\frac{\mathrm{\partial L}}{\mathrm{\partial z}}$. As daily experience shows, dropped objects do not float in mid-air, but accelerate toward the ground, acquiring more and more negative momentum in the vertical direction. Thus momentum is not conserved in this system. (Obviously the momentum of the Earth and even that of the gravitational field have been ignored.) The mere explicit dependence of the Lagrangian upon the location (the coordinate *z*) immediately shows that momentum is not conserved. A translation-invariant Lagrangian (perhaps of particles interacting by instantaneous Newton gravitational forces) could depend on the *difference* of particle positions only.

What about energy conservation? The energy is given by

7 $$ E=-L+\frac{\mathrm{\partial L}}{\mathrm{\partial \dot{z}}}\dot{z}=-\left(\frac{1}{2}m{\dot{z}}^2- mgz\right)+m{\dot{z}}^2=\frac{1}{2}m{\dot{z}}^2+ mgz, $$

an expression familiar even apart from the Lagrangian apparatus. The rate of change of energy is

8 $$ \frac{dE}{dt}=-\frac{\mathrm{\partial L}}{\mathrm{\partial t}}-\dot{z}\left(\frac{\mathrm{\partial L}}{\mathrm{\partial z}}-\frac{d}{dt}\frac{\mathrm{\partial L}}{\mathrm{\partial \dot{z}}}\right)=-\frac{\mathrm{\partial L}}{\mathrm{\partial t}} $$

using the Euler-Lagrange equation. But nothing in the Lagrangian *L* depends *explicitly* on time *t* (other than the dynamical quantities *z* and $\dot{z}$, which are held fixed in the partial differentiation), so $\frac{\mathrm{\partial L}}{\mathrm{\partial t}}=0$. One might call that the temporal uniformity of nature (assumed in this Lagrangian *L*). Physicists speak of the time translation invariance of the Lagrangian (and the laws), because resetting the clock by any fixed amount has no effect on the Lagrangian. The laws of physics are (according to this Lagrangian) the same at all times. Because the Lagrange function is invariant under time translations, energy is conserved.

To set up an analogy with immaterial souls or mental properties that act non-redundantly on the physical world, let us now suppose that *g*, the acceleration due to gravity, is allowed to vary, so one has *g*(*t*). *g* didn’t appear in the momentum, but it did appear in the force, so now the force is time-dependent. Obviously momentum still isn’t conserved; now the rate of change of momentum isn’t constant, either. For such a simple Lagrangian, it is easy to calculate $\frac{dE}{dt}$. One has

9 $$ {\displaystyle \begin{array}{rcl}\frac{dE}{dt}& =& \frac{d}{dt}\left(\frac{1}{2}m{\dot{z}}^2+ mg(t)z\right)=\frac{1}{2}m\frac{\partial \left({\dot{z}}^2\right)}{\mathrm{\partial \dot{z}}}\frac{\mathrm{d}\dot{\mathrm{z}}}{dt}+\frac{\partial \left( mg(t)z\right)}{\mathrm{\partial z}}\frac{dz}{dt}+\frac{\partial \left( mg(t)z\right)\Big)}{dt}\\ {}& =& m\dot{z} \ddot{z}+ mg(t)\dot{z}+m\frac{dg(t)}{dt}z=-\dot{z}\left(- mg(t)-m\ddot{z}\right)+m\frac{dg(t)}{dt}z\\ {}& =& 0+m\frac{dg(t)}{dt}z\ne 0\end{array}} $$

using the Euler-Lagrange equation at the end. Because *g* is not a constant, energy is not conserved. Time translation invariance is violated by the explicit dependence of *g*(*t*) of on *t*.

The role of *g*(*t*) here is a decent toy model of how dualists ought to think of the soul’s (or causally efficacious mental properties’) action upon matter (or material properties): it appears in the equations of motion to affect the motion of matter, but only as an externally applied influence, not as another quantity with its own dynamics based on the principle of least action and hence its own Euler-Lagrange equation(s). Thus the soul makes an indirect contribution to the potential energy of the matter, but it has no mental energy itself (which might involve ${\left(\frac{dg}{dt}\right)}^2$); *mutatis mutandis* the same would hold for mental properties with nonredundant causal influence on matter. These considerations provide support for the claim that all causation of bodily motion involves the transfer of energy. One will be hard-pressed (at least apart from quantum physics) to let the soul affect the body without spoiling energy conservation, because the soul’s effects on the body are time-dependent if they exist at all, and time translation symmetry is equivalent to energy conservation (at least for physics satisfying the principle of least action). A better toy model would treat matter and the mental causal influence as spatially continuous, those imposing an external force that varies with place as well as time. Such a treatment would call for thinking more about how minds might be related to space.

## Minds and Space?

From the standpoint of modern local (and presumably relativistic) field theory, a spatio-temporally varying influence from a non-physical mind is easily accommodated formally. Whether spatio-temporal extension and variation are plausible from the side of the non-physical mind is another matter, albeit one on which I make no claim to expertise.

One should distinguish the question of locality (spatial location and variation) from superficially similar issues in the philosophy of time. As far as classical local field theory is concerned, it would *not* be a problem if someone held the view that, say, irreducibly tensed experience, the nature of language, causation, or some similar considerations required an objective “flow” of time implying absolute simultaneity (Smith 1993; Tooley 1997; Craig 2000; Craig and Smith 2008). While fundamental physics usually studies relativistic local field theory, and relativity provides an additional reasons for field theories to be local, non-relativistic local field theories make sense and are widely used in condensed matter physics. Local field theory is compatible with absolute simultaneity, and such ideas have seen an explosion of attention since 2009 in fundamental physics as well (Hořava 2009). Thus locality can be well motivated even apart from relativity, though relativity provides reasons to think that mental events are spatially located (Russell 1927, p. 384; Weingard^1977^).

Causal influence from nonphysical minds on the physical world having been widely assumed in centuries past, one can gain some useful perspective by attempting to sketch some of that discussion as it addressed the relations of minds to space. As Peter Menzies writes, “[p]hilosophers from Pierre Gassendi onwards have pointed out such causal interaction is impossible within Descartes’ metaphysics which awards primacy to causation by contact forces and in which minds do not have any spatial location or extension.” (Menzies 2013) Unlike vague complaints about mysteriousness or souls and bodies being different and hence causally unrelatable, this complaint about spatiality *vs.* nonspatiality and causation only by contact clearly picks out a real problem. Has physics or dualism (or both) changed so as to ameliorate this problem? Modern physics is not so much by contact as by spatial overlap (at least as a necessary condition). Fields being omnipresent (at least regarding where they are defined, though they could have a value of 0 in some places), the spatial overlap condition is always satisfied. Unlike mechanical bodies, fields do not exclude each other from a location. But as the concept of dark matter reminds us, fields do not automatically influence each other. For two fields to interact directly, each must appear in the other’s equations of motion (a.k.a. “field equations”). For the non-physical to influence the physical, the non-physical must appear in the equations of motion for the physical.

The locality of conservation laws, with many little conservations holding (or not?) in my brain and many little conservations holding elsewhere, makes it important to reconsider where souls are, or at any rate where they act, on the assumption that they exist and act. (The same holds for mental properties that are non-redundantly causally efficacious.) On this point there is a widespread tendency to take Descartes’s dualism as normative or at least representative, despite some features that are distinctive and arguably vulnerable, novel in his day and unpopular among dualists today.

The question of souls’ relationship to space has been discussed, usually negatively. But it is not clear that the reasons are good (Zimmerman 2007; Lycan 2009, 2013; Zimmerman 2010; Bailey et al. 2011; Lycan 2018). Foster’s inference that souls lack spatial location from their being wholly non-physical (Foster 2001) is a bit quick. A substantial part of the doubt about the possibility of Cartesian mind-body interaction (not to mention the pairing problem (Kim 2003)) is due to denying souls spatial location (Wong 2007). According to Jaegwon Kim, “It can be seen that Descartes’s trouble with mental causation has nothing to do with the bruteness or primitiveness of causation or whether causation is merely a matter of Humean regularity, and that it has everything to do with the supposed nonspatiality of Cartesian minds.” (Kim 2007, p. 76)

At least some scholastics had a doctrine that the whole of a soul was present in every part of the space that it occupied (Grant 1981, p. 223). This idea doesn’t seem terribly clear, and was criticized by Henry More, who made spirits more straightforwardly spatial (Grant 1981, p. 223). The views of the scholastics on angels and space were complex, diverse and dynamic (Iribarren and Lenz 2007; Cross 2012), and might be relevant to understanding the tradition of views about human souls’ relations to space (though there was little tendency to think of humans as akin to angels + bodies, as Malebranche perhaps did (Connell 1967)). As a crude summary, one might say that angels were held to be neither fully in space nor fully out of it, with questions about whether presence was merely causal, or substantial (a view generally rejected), or in some other fashion. One might also consider the intelligences who used to drive the planets around (until they were fired due to Copernicanism, the rise of inertia in physics, and the mechanical philosophy (McKaughan and Vande Wall 2018, pp. 252, 283, 565)). Rather than being occasional messengers from a divine realm, these intelligences had full-time jobs relating them to particular places in the physical world.

In the early modern period, *pace* Descartes, some authors such as More and Samuel Clarke did take immaterial souls to be spatially located and to occupy a finite rather than point volume (Vailati 1993; Zimmerman 2007). (Recently doubt has been expressed whether Cartesians really held the views now commonly ascribed to them (Reid 2008).) While More’s and Clarke’s spatially extended souls differ from the scholastic view, all these authors (would have) rejected Descartes’s non-spatial soul interacting with matter at one point in favor of a soul in some fashion related to a region of space (Vailati 1993). Locke ascribed location and mobility to souls, and ascribing finite spatial extent seems to be quite compatible with his views (Brown 2012).

Spatially located souls also address a concern of Lowe’s, who worried that various interactionist theories “seem to be inherently incapable of explaining why the mind’s causal influence upon the material world has to be channelled through the brain: none of them can readily explain why telekinesis is not a ubiquitous phenomenon.” (Lowe 1992) Finite spatial extent also facilitates Eccles’s spatio-temporal patterning of the mind (Eccles 1987). Hasker’s non-Cartesian emergent souls are also spatially located (Hasker 2001). While such views seem to break with dualist tradition, perhaps our view of tradition is faulty. “For even if philosophers today very often take for granted that immaterial entities have no location, this is in fact quite an extraordinary view, historically speaking.” (Pasnau 2011, p. 328)

It is a familiar feature of modern physics (Green’s functions, Dirac delta functions, *etc.* (Jackson 1975)) that if something acts at only one point, achieving a finite effect requires that the act be an infinite spike. This is, perhaps, another reason why dualists should not want to follow Descartes’s point-sized area of the soul’s influence: the soul would have to hit the brain too hard in one area. That would be hard on that part of the brain.

## Conditionality, II

Locality was discussed before conditionality in the preview above, because locality seems easier to describe verbally than does conditionality. However, if one brings in the mathematics of local conservation laws, one might as well include conditionality as well. Thus it makes sense to introduce the mathematics of conditionality in the simplest case with global conservation laws, and only later introduce local (conditional) conservation laws.

Elementary treatments of conservation laws often talk about conservation laws as holding for “closed systems,” a custom widely followed in the philosophy of mind literature (e.g., Montero 2006; Koksvik 2007). Unfortunately it is not terribly clear what it is for a system to be “closed” if one is contemplating immaterial influences. Is an immaterial soul that acts only on the brain, “in” the brain? Must one address the relationship between souls and space? This ambiguity has a simple remedy: drop the talk of closed systems, which rarely appears in advanced physics, and talk instead about symmetries such as time translation invariance and space translation invariance (*a.k.a.* the homogeneity of space), the uniformity of nature. Thus what counts is where a soul acts, not where it is (if it is anywhere).

The conservation of energy and other conserved quantities can be investigated in a simple yet systematic way for classical (that is, not quantum) theories, whether of particles or fields, if the dynamics of the system can be derived from the principle of least action. The action *S* is defined as the time integral of the Lagrangian *L*. For mechanical systems, the Lagrangian is typically the kinetic energy *T* less the potential energy *U*:

10 $$ L{=}_{def}T-U. $$

While Lagrangian dynamics cannot describe every conceivable physical behavior (such as dissipation or quantum behavior), it is the general starting point for fundamental physical theories in contemporary physics. Energy conservation finds its greatest possible warrant in Lagrangian dynamics, so we will meet the objection to dualism in its strongest form while working within Lagrangian dynamics. If the objection fails in a Lagrangian context, then it fails, period.

Conservation can be studied at various levels of mathematical sophistication. Texts on quantum field theory typically address classical field theory in the early chapters. Thus they often begin with Noether’s first theorem, which systematically illuminates the relationship between symmetries (rigid transformations of the description that make no real difference) and conservation laws (Noether 1918; Kaku 1993). This level of mathematical complexity is not necessary for present purposes, however—though Noether’s emphasis on converses to her theorems will be recalled below. Conservation laws in General Relativity, a rather distinctive classical field theory, present still further complexities and perplexities (Anderson 1967), though the latter may be ameliorated somewhat (Pitts 2010).

For now I will recall the advanced undergraduate mechanics derivations of the global conservation of energy and momentum for a collection of particles (e.g., Marion and Thornton 1988). Angular momentum could be treated in much the same way as momentum. The conservation of energy follows if and (basically) only if the Lagrangian lacks explicit dependence on time. The Lagrangian usually depends on time implicitly through dependence on position and/or velocity while still conserving energy, but explicit dependence on time (except of a certain trivial sort) will exclude the conservation of energy. Thus the conservation of energy follows as a *conditional* claim (actually a biconditional one), not a categorical one.

Let us now consider the mathematics. Assume that there are *N* particles indexed by the label *K*, which runs from 1 to *N*, and that these exhaust the physical contents of the world. The *K* th particle has coordinates ${x}_K^i$ and the velocity components ${v}_K^i{=}_{\mathrm{df}}\frac{d{x}_K^i}{dt}$; *i* runs from 1 to 3 because there are (presumably) three spatial dimensions. (This index is more convenient than calling the three coordinates *x*, *y* and *z*. It also extends nicely into relativistic physics. When relativity and space-time are in view, it is customary to use a superscript rather than a subscript as the index for coordinates, hence *x*^*i*^ rather than *x*_*i*_.) The energy function involves summing over all the *N* particles and over all of their three spatial coordinates:

11 $$ E=-L+\sum \limits_{K=1}^N\sum \limits_{i=1}^3\frac{\mathrm{\partial L}}{\partial {v}_K^i}{v}_K^i. $$

The conservation of energy would be just the vanishing (being 0) at all times of the total time derivative $\frac{dE}{dt}$. The total time derivative includes both explicit time dependence, which would arise from an external influence, in this case including any immaterial entities acting on the *N* particles (which will appear in the term $\frac{\mathrm{\partial L}}{\mathrm{\partial t}}$, the partial derivative with respect to *t* when the velocities ${v}_K^i$ and coordinates ${x}_K^i$ are held constant), and implicit time dependence through the positions and velocities of the *N* bodies. As will appear, explicit time dependence will spoil conservation. (In the interest of compactness, quantifiers over time will be suppressed for a while.)

12 $$ {\displaystyle \begin{array}{rcl}\frac{dE}{dt}& =& \frac{d}{dt}\left(-L+\sum \limits_{K=1}^N\sum \limits_{i=1}^3\frac{\mathrm{\partial L}}{\partial {v}_K^i}{v}_K^i\right)\\ {}& =& -\frac{\mathrm{\partial L}}{\mathrm{\partial t}}-\sum \limits_{K=1}^N\sum \limits_{i=1}^3{v}_K^i\left(\frac{\mathrm{\partial L}}{\partial {x}_K^i}-\frac{d}{dt}\frac{\mathrm{\partial L}}{\partial {v}_K^i}\right).\end{array}} $$

The expressions $\frac{\mathrm{\partial L}}{\partial {x}_K^i}-\frac{d}{dt}\frac{\mathrm{\partial L}}{\partial {v}_K^i}$ are just the Euler-Lagrange derivatives of the Lagrangian. The Euler-Lagrange equations of motion assert that these derivatives are 0: for each particle *K* and coordinates *i*,

13 $$ \frac{\mathrm{\partial L}}{\partial {x}_K^i}-\frac{d}{dt}\frac{\mathrm{\partial L}}{\partial {v}_K^i}=0. $$

These equations of motion are equivalent to Newton’s laws when Newtonian and Lagrangian formulations both apply. The Euler-Lagrange equations look like Newton’s laws in terms of the momenta ${p}_{\mathrm{iK}}=\frac{\mathrm{\partial L}}{\partial {v}_K^i}$ in simple cases, such as if the Newtonian force is just the (negative of the) derivative of the potential energy and the coordinates are Cartesian:

14 $$ \frac{\mathrm{\partial L}}{\partial {x}_K^i}-\frac{d}{dt}\frac{\mathrm{\partial L}}{\partial {v}_K^i}=\frac{\partial \left(T-U\right)}{\partial {x}_K^i}-\frac{d}{dt}{p}_{\mathrm{iK}}=\frac{\partial \left(-U\right)}{\partial {x}_K^i}-\frac{d}{dt}{p}_{\mathrm{iK}}=0. $$

This is basically Newton’s second law for the *K* th particle in the form *F* − *ma* = 0. If one adds these equations for all particles, the total momentum is conserved if and only if the position of the center of mass does not enter the Lagrangian. Then the Lagrangian is independent of the position of the system as a whole, depending only on the relative coordinates such as ${x}_1^i-{x}_2^i$. The Lagrangian is translation-invariant; space is homogeneous. That the conservation of momentum follows from the invariance of the Lagrangian under spatial translations was understood by Hamiltonian and Jacobi in the 1830s (Hamilton 1834; Jacobi 1996). But it was not generally accepted that physics should use a variational principle until perhaps the 1920s. (Some sources on the history of the relationship between symmetries and conservation laws exist (Houtappel et al. 1965; Kastrup 1987; Kosmann-Schwarzbach 2011; Pitts 2016).)

The conservation of angular momentum follows from a similar argument. If the Lagrangian is invariant under a rotation of all the particles together, then space is isotropic. Isotropy underlies the conservation of angular momentum much as invariance under time translation underlies the conservation of energy and spatial homogeneity underlies the conservation of momentum. Momentum conservation and perhaps angular momentum conservation could constitute objections to dualism in the same way that energy conservation does.

Returning to question of the conservation of energy, one sees that, using the Euler-Lagrange equations, energy is conserved if and (more or less) only if the Lagrangian does not depend explicitly on time:

15 $$ \frac{dE}{dt}=-\frac{\mathrm{\partial L}}{\mathrm{\partial t}}-\sum \limits_{K=1}^N\sum \limits_{i=1}^3{v}_K^i\left(\frac{\mathrm{\partial L}}{\partial {x}_K^i}-\frac{d}{dt}\frac{\mathrm{\partial L}}{\partial {v}_K^i}\right)=-\frac{\mathrm{\partial L}}{\mathrm{\partial t}} $$

for solutions of the equations of motion. If $\frac{\mathrm{\partial L}}{\mathrm{\partial t}}\ne 0$ (and if one cannot remove this dependence by adding a total time derivative to the Lagrangian, an addition which has no effect on the Euler-Lagrange equations of motion), then at least some of the particles experience a time-dependent influence. Then energy conservation fails. Assuming $\frac{\mathrm{\partial L}}{\mathrm{\partial t}}=0$ (the manifest form of the time-translation invariance of the Lagrangian and the physical laws), then one has energy conservation:

16 $$ \left(\forall t\right)\left(\frac{dE}{dt}=0\right), $$

a result known already to Lagrange over two centuries ago (1997). Now the universal quantifier over time *t* is explicit, partly to pump intuitions about what the failure of conservation would mean logically in terms of de Morgan’s law $\neg \left(A\&B\right)\iff \left(\neg A\vee \neg B\right)$ and its quantifier analog, partly to help to prepare for the treatment of continuous matter below, in which time and space are treated more symmetrically. All the equations above were tacitly universally quantified over all times.

## Locality, II

Consider *N* fields *ϕ*_*K*_(*t*,*x*,*y*,*z*), real functions of position and time, and let *K* be an index that runs from 1 to *N*. The fields can include vector fields as in electromagnetism and the weak and strong nuclear forces, tensor fields as in the case of gravity, spinor fields for electrons, *etc.*, by treating each component as a field (and taking real and imaginary parts or the like for complex fields); this derivation aims to be mathematically simple and capacious. Whereas in the previous derivation, time was the independent variable on which the positions and hence the velocities depended, now the time and space are the independent variables on which the fields *ϕ*_*K*_ and their time and space derivatives $\frac{\partial {\phi}_K}{\mathrm{\partial t}}$ and $\frac{\partial {\phi}_K}{\partial {x}^i}$ depend.

Conservation laws for continuous matter/fields, using the principle of least action, are derived from a Lagrangian *L* defined as the spatial volume integral of the Lagrangian density $\mathcal{L} :$

17 $$ L={\int}_{all\  space}{d}^3x\mathcal{L} ={\int}_{-\infty}^{\infty } dx{\int}_{-\infty}^{\infty } dy{\int}_{-\infty}^{\infty } dz\mathcal{L} . $$

The Lagrangian density $\mathcal{L}$ is a function of *ϕ*_*K*_(*t*,*x*^*i*^), their time and space derivatives $\frac{\partial {\phi}_K}{\mathrm{\partial t}}\left(t,{x}^i\right)$ and $\frac{\partial {\phi}_K}{\partial {x}^i}\left(t,{x}^i\right)$ and maybe time *t* and place *x*^*i*^. In the early 1910s Max Born expressed how rigid translation symmetries imply conservation laws:

The assumption of Mie just emphasized, that the function [$\mathcal{L}$] is independent of *x*, *y*, *z*, *t*, is also the real mathematical reason for the validity of the momentum-energy-law. … We assert that for these differential equations, a law, analogous to the energy law (3${}^{\prime }$) of Lagrangian mechanics, is always valid as soon as one of the 4 coordinates *x*_*α*_ does not appear explicitly in [$\mathcal{L}$]. (Born 1914)

The relevant mathematics is well understood and available in many graduate-level textbooks (e.g., Goldstein 1980, chapter 12; Davis 1970). Readers not interested in the mathematics are welcome to skip the equations that lack explicit quantifiers.

The previous section already illuminated the logical status of conservation laws as conditional claims using the simplest adequate mathematics: a world with *N* particles, possibly subject to external (immaterial) influences. Now let us see how the same issues arise with the further feature that conservation laws are primarily local rather than global. This effort will require multi-variable rather than single-variable differential calculus, to discuss how a quantity dependent upon several others changes when one of the independent variables changes and the others remain the same. Local conservation laws state that for each small region of space, the amount of energy (or momentum, angular momentum, or the like) changes over time only because some of the conserved quantity flows out through the boundaries of the region; more mathematically, the density *ρ* of the conserved quantity and its current density *J*^*i*^ satisfy

18 $$ \left(\forall t\right)\left(\forall x\right)\left(\forall y\right)\left(\forall z\right)\left(\frac{\mathrm{\partial \uprho}\left(t,x,y,z\right)}{\mathrm{\partial t}}+\sum \limits_{k=1}^3\frac{\partial {J}^i\left(t,x,y,z\right)}{\partial {x}^i}=0\right). $$

One can group time and space together using Greek indices running from 0 (time) to 3): *x*^*ι*^ = 〈*t*,*x*^*i*^〉. (Using coordinates with an index is advantageous over bold-faced vector notation, because the former but not the latter also naturally works for General Relativity.) The Euler-Lagrange field equations follow from the Lagrangian density $\mathcal{L} \left({\phi}_K,\frac{\partial {\phi}_K}{\partial {x}^{\iota }},{x}^{\iota}\right)$. The explicit dependence on *x*^*ι*^ permitted here leaves room for influence from immaterial substances to avoid begging the question against dualism. For brevity I will write partial derivatives $\frac{\partial {\phi}_K}{\partial {x}^{\iota }}$ as *ϕ*_*K*_,_*ι*_ and the like. If we write *ρ* as *J*^0^, then the continuity equation for conservation is

19 $$ {\displaystyle \begin{array}{rcl}\left(\forall t\right)\left(\forall x\right)\left(\forall y\right)\left(\forall z\right)\left(\frac{\mathrm{\partial \uprho }}{\mathrm{\partial t}}+\sum \limits_{k=1}^3\frac{\partial {J}^i}{\partial {x}^i}=0\right)& =& \left(\forall t\right)\left(\forall x\right)\left(\forall y\right)\left(\forall z\right)\left(\sum \limits_{\iota =0}^3\frac{\partial {J}^{\iota }}{\partial {x}^{\iota }}=0\right)\\ {}& =& \left(\forall t\right)\left(\forall x\right)\left(\forall y\right)\left(\forall z\right)\left(\sum \limits_{\iota =0}^3{J}^{\iota }{,}_{\iota }=0\right).\\ {}& & \end{array}} $$

This kind of equation holds for each quantity that is locally conserved, such as energy, momentum and angular momentum are if the appropriate symmetries hold. The continuity equation expresses a local conservation law.

For the conservation of energy and momentum (if in fact they are conserved), one gets a total of four continuity equations at each space-time point. The canonical energy-momentum “tensor” (which can fail to be a tensor in some cases, such as General Relativity (Anderson 1967; Wald 1984; Pitts 2010) is

20 $$ {T}_{\nu}^{\iota }=-\mathcal{L} {\delta}_{\nu}^{\iota }+\sum \limits_{K=1}^N\frac{\partial \mathcal{L}}{\partial {\phi}_K{,}_{\iota }}{\phi}_K{,}_{\nu }, $$

where ${\delta}_{\nu}^{\iota }$, the “Kronecker *δ*”, is 1 if *ι* = *ν* and 0 otherwise. If desired, one can break up this equation into the energy and momentum pieces. Setting *ν* = 0, one gets the expression for energy:

21 $$ {T}_0^{\iota }=-\mathcal{L} {\delta}_0^{\iota }+\sum \limits_{K=1}^N\frac{\partial \mathcal{L}}{\partial {\phi}_K{,}_{\iota }}{\phi}_K{,}_0; $$

thus the first term is present for the energy density ${T}_0^0$ but vanishes for the energy flux ${T}_0^i$. Setting *ν* = *n* (*n* = 1 to 3), one gets the expression for momentum:

22 $$ {T}_n^{\iota }=-\mathcal{L} {\delta}_n^{\iota }+\sum \limits_{K=1}^N\frac{\partial \mathcal{L}}{\partial {\phi}_K{,}_{\iota }}{\phi}_K{,}_n; $$

thus the first term vanishes for the momentum density ${T}_n^0$ but is present for the diagonal components (*i* = *n*, pressure) of the momentum flux ${T}_n^i$.

Returning now to the 4-dimensional expressions for streamlining, we can now ascertain the circumstances under which energy and momentum are locally conserved by taking the total four-divergence of the canonical energy-momentum tensor, including both explicit dependence on *x*^*ι*^ (which could include the influence of immaterial entities) and implicit dependence on the space-time location through the field *ϕ*_*K*_ and its spatio-temporal partial derivatives:

23 $$ {\displaystyle \begin{array}{rcl}\sum \limits_{\iota =0}^3\frac{d}{d{x}^{\iota }}{T}_{\nu}^{\iota }& =& \sum \limits_{\iota =0}^3\frac{d}{d{x}^{\iota }}\left(-\mathcal{L} {\delta}_{\nu}^{\iota }+\sum \limits_{K=1}^N\frac{\partial \mathcal{L}}{\partial {\phi}_K{,}_{\iota }}{\phi}_K{,}_{\nu}\right)\\ {}& =& -\frac{\partial \mathcal{L}}{\partial {x}^{\nu }}-\sum \limits_{K=1}^N{\phi}_K{,}_{\nu}\left(\frac{\partial \mathcal{L}}{\partial {\phi}_K}-\sum \limits_{\iota =0}^3\frac{d}{d{x}^{\iota }}\frac{\partial \mathcal{L}}{\partial {\phi}_K{,}_{\iota }}\right).\end{array}} $$

The expression in parentheses is the Euler-Lagrange derivative of the Lagrangian density for the *K* th field *ϕ*_*K*_; the field equations just assert that this quantity vanishes (for each field and each space-time point),

24 $$ \frac{\partial \mathcal{L}}{\partial {\phi}_K}-\sum \limits_{\iota =0}^3\frac{d}{d{x}^{\iota }}\frac{\partial \mathcal{L}}{\partial {\phi}_K{,}_{\iota }}=0. $$

Using the field equations, one therefore has

25 $$ \left(\forall t\right)\left(\forall x\right)\left(\forall y\right)\left(\forall z\right)\left(\sum \limits_{\iota =0}^3\frac{d}{d{x}^{\iota }}{T}_{\nu}^{\iota }=-\frac{\partial \mathcal{L}}{\partial {x}^{\nu }}\right). $$

For *ν* = 0 this is just the local conservation of energy (if the right side is 0 at the place (*t*,*x*,*y*,*z*) in question); for *ν* = 1,2,3 it is the local conservation of momentum (again, if the right side is 0 there and then). Locality and conditionality together have the consequence that wherever and whenever no immaterial influence acts on matter, then energy and momentum are conserved. If the right side $-\frac{\partial \mathcal{L}}{\partial {x}^{\nu }}$ is nonzero in some parts of space-time, then energy and/or momentum fails to be conserved in those parts. For present purposes, the most plausible parts would be those times and places where brains of living persons are.

The canonical energy-momentum ‘tensor’ defined above arguably has certain vices, such as not being symmetric in many cases (symmetry being useful to define angular momentum) and depending on arbitrary choices (gauge-dependence) in electromagnetism. These can be fixed by Belinfante’s symmetrization procedure and are irrelevant for present purposes. There are additional conceptual problems in relation to General Relativity, some of which make it a distinctive theory requiring special treatment. The above is a no-frills treatment of the core ideas needed for present purposes, foregrounding the relationship between symmetry and conservation with the simplest adequate mathematics.

One can relativize conservation laws and our warrant for accepting them to places. Even some of the best work (Papineau 2000) speaks of conservation of energy in the singular, as though there were just one bit of accounting to do, rather than uncountably infinitely many. If there is a singular conservation of energy to talk about, it is presumably either a global law, or a vast conjunction of all the local conservations. The former is obtained by integrating (adding up) all the local conservation laws and is logically too weak; the subterfuge of nonlocal compensation exploits this weakness. Global conservation laws, moreover, can fail to make mathematical sense if the matter of the universe doesn’t think out fast enough at large distances (Peebles 1993, p. 139). The other singular entity that one might envisage for the conservation of energy is a conjunction of all the local conservation laws. However, its warrant is only as great as that of its weakest link. If the question of energy conservation *in the brain* is being discussed, then our warrant for this conjunctive conservation law is no better than our warrant for conservation in the brain (assuming that to be the weakest link).

## Not Begging the Question: Neuroscience?

Given experiences testing the conservation of energy in secondary school or in university science laboratories, one might think that it is straightforward to ascertain empirically whether energy is conserved. But at least in fundamental physics it is difficult to test conservation laws directly. That is because the mathematical expression for energy density and energy flux density (and likewise for momentum and for angular momentum) is highly theory-dependent. It also involves performing a spatial integral of an expression (typically) quadratic in first derivatives of the physical fields—which is extremely different from sticking a thermometer into a beaker (though thermometers themselves are striking theory-rich achievements (Chang 2007)). It is more reasonable simply to test (empirically) whether the theory’s equations of motion hold and (theoretically) whether the theory is time translation-invariant. While sufficiently gross violations wouldn’t require the mathematics to discern—such as if an aircraft carrier suddenly appeared in a wheat field in Kansas *ex nihilo*, or even levitation (controversially ascribed to 19th century medium Daniel D. Home and 17th century (St.) Joseph of Copertino (Dingwall 1947; Braude 1986; Grosso 2016), though Braude thinks that conservation still holds, as noted above)—there seem not to be such cases pertaining to the philosophy of mind. Admittedly a microscopic field description of the brain might be unnecessary; some macroscopic description might suffice. But such a description will involve detailed consideration of the structure and function of the brain: neuroscience.

What kind of evidence is required to count against dualism? Wade Savage claimed against Eccles (1976) that it was unnecessary to look at the brain to reject dualism, even though the extant empirical evidence didn’t rule dualism out and perhaps evidence never would (Savage 1976); the “mysterious and inexplicable” nature of immaterial mental influence on the physical sufficed. A contrary view is that looking at the brain is clearly the most relevant kind of evidence and there has not been enough of it to dismiss dualism (Foster 1989; Meixner 2005; Koksvik 2006; Thompson 2008; Garcia 2014; White 2017). Many neuroscientists would disagree that there has not been enough evidence from the brain. Perhaps larger world-view issues of intellectual history, not just detailed micro-argumentation characteristic of analytic philosophy, play a crucial role in such judgments (Evans 1981; Sturgeon 1998; Burge 1993, p. 360). The rapidity with which words like “ghosts,” “spooks” and “superstition” are deployed and the ease with which dualism is dismissed with what Lycan calls weak arguments (2009) suggest that many feel that it should not be necessary to argue about such matters nowadays. Coherence between dualism and theism has also been suggested by people on both sides of the theistic-nontheistic divide (Bunge 1980; Taliaferro 1994; McGinn 1999; Foster 2001; Meixner 2004; Plantinga 2007). Whether or not it is too early to dismiss dualism given the evidence — a difficult question if Bayesian priors are subjective — evidence from the brain (past or future) is especially relevant. Precious little such evidence was available to Leibniz, for example. Fortunately some detailed empirical investigation of such matters in fact has been occurring (Wilson 1977; Wilson 1993; Wilson 1995; Wilson 1999; McDermott 2001; Clarke 2009; Clarke 2014). Here is an example (Wilson 1999):

If mind is not a part of the physical universe but is able to influence brain events, then violations of physical laws should occur at points of such mental influence. Using current knowledge of how the nervous system functions, the minimal necessary magnitude of such violations is examined.… A variety of influences that could produce action potentials is considered, including the direct opening of sodium channels in membranes, the triggering of release of neurotransmitter at synapses, the opening of postsynaptic, ligand-gated channels, and the control of neuromodulation. It is shown that the magnitude of the disturbance required is significantly greater than allowed for under quantum-mechanical uncertainty. It is concluded that violations of fundamental physical laws, such as energy conservation, would occur were a non-physical mind able to influence brain and behaviour.

This is a refreshingly detailed critique, though one might worry that the Leibnizian energy conservation objection is sneaking back in (which is fair for Wilson’s purpose but not for ours). Peter Clarke’s work also exemplified the right sort of critique (Clarke 2009, 2010a, 2010b, 2014). According to Clarke, a neuroscientist (now deceased) and evidently a Christian but a dual-aspect monist, [m]odern substance dualist philosophers continue to argue that their views are compatible with neuroscience, and I think that part of the reason is that they pay careful attention only to neuropsychology, largely ignoring the strong mechanistic implications of the other branches of neuroscience. When evidence from the whole breadth of neuroscience is taken together, it constitutes a truly formidable challenge to substance dualism. In my opinion the only kind of substance dualism that is still even remotely defendable in the light of modern neuroscience is a limited one, invoking a separate soul acting on the brain only for very particular aspects of our humanity such as free will (e.g. the philosopher Robert Kane) (Clarke 2009).

In contrast to the question-begging brain-free argument considered above, Clarke criticizes dualism for a right kind of reason. Papineau also stresses empirical considerations from neuroscience (Papineau 2000, pp. 202, 203). I suggest that those looking to criticize dualism for reasons that ought to be persuasive (rather than question-begging) look to neuroscience, where, apparently according to most experts, such reasons will be found.

## Conclusion

To sum up, the energy conservation objection generally discussed from the 17th to the 21st centuries, being question-begging, has little to recommend it, even without drawing upon quantum mechanics (which might yield further loopholes). At best the objection provides a way to frame an incredulous stare more articulately while sounding pro-science. When one opens the black box of conservation laws, one finds a mirror reflecting back one’s own beliefs. Some of the traditional responses by dualists are perhaps even less impressive, betraying lack of awareness of what conservation laws say and where they come from. However, the (bi)conditionality response is deeply rooted in the most relevant physics, Noether’s first theorem and its converse, and had an important role in the original 18th century debate as well.

Two related challenges to dualism are more worth attention, namely, the *a posteriori* empirical question from neuroscience and the novel difficulty posed by General Relativity (Pitts 2019b); the latter might intersect in interesting ways with larger worldview considerations.

## Footnotes

1 See also (Penrose 1994, pp. 334, 344-346) for a related suggestion that this peculiarity of General Relativity might help to address a conservation-related difficulty of GRW spontaneous collapse theories. Penrose invokes gravity to induce collapse of the wave function. This project is also linked to the philosophy of mind.

2 I thank an anonymous referee for clarity on this point.

3 This answer seems to have been fairly common in 18th century Germany, such as in Knutzen and Crusius (Watkins 1995; Watkins 1998).

4 One can see Euler as a forerunner of naturalistic metaphysics in another area, *viz.* his claim that the laws of mechanics are so certain that a metaphysics of nature must be answerable to mechanics, not vice versa (Breidert 2007) as the more traditional Wolffian claim held (Euler 1750).

5 One would be hard-pressed to express in scientific language any overdetermination claims to the effect that the soul only tends to cause events that would have happened anyway, or the like. In the current philosophy of mind literature, causal closure can mean many things (Montero 2003) and at least some dualists manage to affirm at least some versions (Lowe 2003). Such claims seem like a retreat for the dualist, however.
